# Supplementary material for: Prevalence and aeroallergen sensitization in pediatric Allergic Rhinitis: A population-based study in Jeju, Korea
Source: PLoS One. 2025 Jun 13;20(6):e0326070. doi: 10.1371/journal.pone.0326070 (PMC12165365; doi:10.1371/journal.pone.0326070)
Supplement: Attachment — Submitted filename: KOREAN AR PLOS ONE.docx [file pone.0326070.s002.docx]

IT IS A VERY INTERESTING WORK. I BELIEVE IT CAN IMPROVE BY CONSIDERING THE FOLLOWING NOTES

34 significantly associated with increased AR risk (OR 1.58, p=0.042)

INCLUDE THE CONFIDENCE INTERVALS.

61 Jeju Island exemplifies this issue. Despite comprising only 1% of Korea’s population. INCLUDE THE TOTAL POPULATION OF KOREA

78 A total of 1,918 subjects were recruited from 13 public schools in Jeju, South Korea, between..

IT DOES NOT SPECIFY HOW THE SAMPLING AND POPULATION SELECTION WERE PERFORMED

81 results, a total of 1,067 students were included in the final analysis (mean age, 12.5 ± 2.6 82 years; median age, 13.0; interquartile range [IQR], 10–16). The number of girls was similar 83 to the number of boys (male: female ratio, 1.00:1.08) among the 1,067 students. Among 84 those, 473 (44%) subjects were aged 9 to 11 years and came from 6 elementary schools, 322 85 (30%) subjects were aged 13 to 14 years and came from 3 middle schools, and 272 (26%) 86 subjects were aged 16 years and came from 4 high schools.

THIS INFORMATION SHOULD BE INCLUDE IN THE RESULTS SECTION.

93 distributed by teachers at the participating schools. Prior to completing the questionnaire, all

94 participants in the study provided informed consent.

WHO SIGN THE INFORMED CONSENT THE PARTICIPANS OR THEIR PARENTS ¿

97 did not have a cold or the flu?” After completing the form, the questionnaire was taken back 98 to the schools for collection.

IF THE QUESTIONNAIRES WERE ANSWERED BY THE PARENTS ¿ PLEASE CLARIFY THAT THE ISAAC METHODOLOGY WAS USED AND PROVIDE THE REFERENCE.

106 added. Lengths and widths of wheals were measured 15 minutes following application. 107 Sensitization to an antigen was defined as a mean wheal size that was the same size as or 108 larger than that of the positive control (allergen/histamine ratio ≥1). A skin test was 109 considered invalid if the wheal size for the positive control (1% histamine) was smaller than 110 2 mm or if a wheal was observed in the negative control (normal saline 0.9%).

IS THIS TECHNIQUE PROPIETARY OR AN INTERNATIONALTECHNIQUE, PROVIDE THE REFERENCE.

115 aeroallergens. The number of positive skin test responses was also estimated as the 116 percentage of the subjects among the sensitized subjects.

120 (monosensitized or polysensitized) on the prevalence of AR was evaluated using univariate

121 and multivariate logistic regression analyses.

PLEASE SPECIFY THE CRITERIA USED TO DETERMINE WHICH VARIABLES WERE INCLUDE IN THE MULTIVARIATE ANALYSIS.

124 Results	HERE THE INFORMATION OF LINES	81 -85 SHOULD BE PRESENTED

100 common aeroallergens (D.pteronyssinus, D.farinae, JC, pine, willow, maple, birch, oak, alder, 101 timothy grass, Bermuda grass, ragweed, mugwort, Japanese hop, Penicillium, Aspergillus, 5 102 Cladosporium, and Alternaria) and 2 controls (positive and negative) were administered to 103 all subjects

WHY WERE COMMON PETS SUCH AS CATS AND DOGS NOT INCLUDED IN THE STUDY AS THEY ARE IN OTHER STUDIES ¿

149 Fig 2. Categorization of schoolchildren using allergy skin test responses and the ISAAC 150 questionnaire (n = 1,067)

I RECOMMEND THIS TYPE OF TABLE IT IS MORE READABLE AND EASIER TO UNDERSTAND

|  |  | **ISAAC** | |  |
| --- | --- | --- | --- | --- |
|  |  | **ISAAC +** | **ISACC -** |  |
| **SKIN TEST** | S. TEST + | 309 | 173 |  |
|  | S. TEST - | 241 X% | 344 |  |
|  | TOTAL |  |  |  |
|  |  |  |  |  |

177 Table 3 presents the characteristics associated	with AR

THE STUDY WAS DESIGNED AS AN ASSOCIATION MODEL, BUT IT IS PRSESENTED AS A PREDICTIONMODEL FOR AR.

178 sensitization to dust mites was a significant risk factor for AR (odds ratio [OR]: 1.66; 95% 179 confidence interval [CI]; 1.064-2.580; p = 0.025)

THE FOLLOWING FORMAT IS RECOMMENDED

AR . OR: 1.66; (95% CI; 1.064-2.580); p = 0.025

184 Table 3. Unadjusted and adjusted ORs for the independent predictors of AR 185 development among the sensitized (n = 550)

PLEASE CLARIFY THAT IS THE CRUDE AND ADJUSTED MUTIVARIED MODEL.

277 significant risk factor for AR (p = 0.042) [10],

THE OR QUANTIFYNG THE RISK FACTOR SHOUL BE PLACED BEFORE DE p-VALUE

IN THE DISCUSSION THE FINDINGS ON SENSITIZATION IN TROPICAL AREAS SHOULD BE PRESENTED IN A CONCRETE MANNER
