# Supplementary material for: Prevalence and aeroallergen sensitization in pediatric Allergic Rhinitis: A population-based study in Jeju, Korea
Source: PLoS One. 2025 Jun 13;20(6):e0326070. doi: 10.1371/journal.pone.0326070 (PMC12165365; doi:10.1371/journal.pone.0326070)
Supplement: Attachment — Submitted filename: Response to Reviewer_2.docx [file pone.0326070.s005.docx]

**Rebuttal Letter**

Dear Reviewer 2,

Dr. Song and colleagues reported on the prevalence and aeroallergen sensitization in pediatric allergic rhinitis based on a population-based study in Jeju, Korea.

In epidemiological research, unique regional allergy data are valuable as they provide important insights into the onset and increase of allergies. This study uses data from eight years ago, but it would be helpful to explain why it was published at this timing.

Thank you for this insightful and understanding comment. We acknowledge that the data collection was conducted eight years ago (2016). Initially, we submitted this manuscript for publication twice—in 2018 and again in 2019—but after receiving rejections both times, we became somewhat discouraged and delayed resubmission. However, considering the unique epidemiological insights provided by our data, particularly the region-specific sensitization patterns in Jeju, we believe the findings remain highly relevant and valuable even today. Your supportive and constructive feedback has greatly encouraged us, and we are sincerely grateful to the reviewers for helping us improve and ultimately publish this important work.

The results and discussion include findings on Japanese cedar; however, the sensitization rate appears to be lower than that reported in Japanese epidemiological studies. Please consider citing the following paper and discussing the differences:

Kiguchi T, et al. Pollen-food allergy syndrome and component sensitization in adolescents: A Japanese population-based study. PLoS One. 2021 Apr 14;16(4):e0249649.

We sincerely thank the reviewer for this insightful suggestion. We have now included the recommended paper by Kiguchi et al. (2021), which provides valuable recent epidemiological data on Japanese cedar pollen sensitization. **We referenced this study in the discussion section to emphasize the notable differences between sensitization patterns in Jeju and those observed in Japan.** Specifically, we highlighted that Kiguchi et al. reported JC pollen as the most prevalent allergen (78.5%) among 13-year-old adolescents with current rhinitis living in Tokyo between 2017 and 2019. In contrast, our study observed that JC pollen ranked third in prevalence after dust mites (*D. pteronyssinus* and *D. farinae*) among Jeju schoolchildren. **These differences likely reflect distinct regional environmental exposures and allergen profiles, underscoring the importance of region-specific allergen monitoring and preventive strategies.**

We appreciate your suggestion, as this comparison significantly enhances the contextual interpretation of our findings.

🡺 Comparative analyses further underscore the distinct sensitization profile in Jeju. In a Japanese study (2006–2007), JC pollen was reported as the predominant allergen (56%) [24]. Another Japanese study (2017-2019) similarly found JC pollen to be the most prevalent allergen, with a sensitization rate of 78.5% among 13-year-old adolescents with current rhinitis living in Tokyo [28]. In contrast, the sensitization pattern in Jeju was dominated by dust mites (*D. pteronyssinus* and *D. farinae*), while JC pollen ranked third. Furthermore, a previous Korean study involving 2,991 schoolchildren with AR also identified *D. pteronyssinus* (76.8%) and *D. farinae* (68.1%) as the leading allergens, followed by birch pollen (10.8%) [14]. These regional differences highlight the necessity of considering geographic and climatic factors when interpreting allergen sensitization data.

Line 34: Since only the odds ratio is presented, please add the 95% confidence interval.

Line 50-51: Please remove the terms "in vitro" and "in vivo."

Line 57: The term "smaller" is unclear—please specify what it is being compared to.

Line 78: Please add the study design at the beginning of the Methods section.

Line 131: Should this be a "Table" instead of a "Figure"?

Line 34: Since only the odds ratio is presented, please add the 95% confidence interval.

significantly associated with increased AR risk (OR 1.58, p=0.042)

🡺 significantly associated with increased AR risk (OR 1.58, 95% CI: 1.017–2.455, p=0.042).

Line 50-51: Please remove the terms "in vitro" and "in vivo."

Diagnosis is based on clinical history and physical examination with sensitization confirmed through *in vivo* skin prick tests or *in vitro* serologic analyses of IgE antibodies [8].

🡺 Diagnosis is based on clinical history and physical examination with sensitization confirmed through skin prick tests or serologic analyses of IgE antibodies [8].

Line 57: The term "smaller" is unclear—please specify what it is being compared to.

While national surveys have provided valuable insights into sensitization patterns [10-15], they often fail to capture the unique characteristics of regional populations with smaller population sizes, where environmental and socio-economic factors can lead to distinct patterns of allergen exposure and sensitization. As a result, national-level sensitization data may have limited applicability in certain regions.

Jeju Island exemplifies this issue. Despite comprising only 1% of Korea’s population, Jeju reports the highest AR prevalence among the nation’s 17 administrative districts [16].

🡺 While national surveys have provided valuable insights into sensitization patterns [10-15], national-level sensitization data may have limited applicability in smaller, environmentally distinct populations compared to large-scale, national populations. Jeju Island exemplifies this issue. Despite comprising only 1**.27**% of Korea’s total population **(**approximately 6**5** million in Jeju out of 51 million nationwide as of 2017 [16]), Jeju reports the highest AR prevalence among the nation’s 17 administrative districts [17].

The term "smaller" was intended to refer to populations smaller than those typically represented in national surveys, where local environmental and socio-economic characteristics could lead to unique patterns of allergen exposure and sensitization. To clarify this point, **we revised the sentence as above.**

**This revision explicitly clarifies the intended comparison between smaller, localized populations and the larger-scale populations typically represented in national surveys**

Line 78: Please add the study design at the beginning of the Methods section.

78 A total of 1,918 subjects were recruited from 13 public schools in Jeju, South Korea, between.

IT DOES NOT SPECIFY HOW THE SAMPLING AND POPULATION SELECTION WERE PERFORMED

Thank you for your insightful comment. We added the lines about the sampling and population selection to the paragraph for further clarification.

🡺 Jeju Island is **geographically** divided by Mt. Halla into **two** main **administrative** regions: **Jeju City in the north and Seogwipo City in the south.** Historically, human settlements have developed **along coastal areas** where spring water sources (Yongcheonsu) are available. **Currently, approximately 75% of Jeju's population resides in Jeju City in the north**. Considering the geographic balance, we aimed to include **schools evenly distributed across the northern and southern regions**. Subjects were ultimately recruited from schools that were cooperative and agreed to participate in our research, as shown in Figure 1. *A total of 1,918 subjects were recruited from 13 public schools in Jeju, South Korea, between.*

Fig. 1 The approximate geographical locations of the selected schools (circles): six elementary schools (green), three middle schools (blue), and four high schools (red).

Line 131: Should this be a "Table" instead of a "Figure"?

Thank you for pointing out this potential source of confusion. In this context, the term "figures" refers specifically to numerical values, not graphical figures. To avoid any misunderstanding, **we have revised the sentence clearly as follows:**

"**Numbers** in parentheses indicate percentages."

We appreciate your careful review, which helped us clarify this point.
